# Genomic origin of *Citrus reticulata* “Unshiu”

**DOI:** 10.1093/hr/uhaf015

**Published:** 2025-05-01

**Authors:** Shengjun Liu, Luoyun Wang, Xiang Zhang, Lifang Sun, Fuzhi Ke, Yue Huang, Lizhi Song, Haiping Ye, Jianguo Xu, Yuantao Xu, Xia Wang, Xiuxin Deng, Gaoping Liu, Qiang Xu

**Affiliations:** National Key laboratory for Germplasm Innovation & Utilization of Horticultural Crops, Huazhong Agricultural University, Shizishan street No.1, Wuhan 430070, China; The Citrus Research Institute of Zhejiang Province, Zhejiang Academy of Agriculture Sciences, Yushanping street No.1, Taizhou 318020, China; National Key laboratory for Germplasm Innovation & Utilization of Horticultural Crops, Huazhong Agricultural University, Shizishan street No.1, Wuhan 430070, China; The Citrus Research Institute of Zhejiang Province, Zhejiang Academy of Agriculture Sciences, Yushanping street No.1, Taizhou 318020, China; The Citrus Research Institute of Zhejiang Province, Zhejiang Academy of Agriculture Sciences, Yushanping street No.1, Taizhou 318020, China; National Key laboratory for Germplasm Innovation & Utilization of Horticultural Crops, Huazhong Agricultural University, Shizishan street No.1, Wuhan 430070, China; National Key laboratory for Germplasm Innovation & Utilization of Horticultural Crops, Huazhong Agricultural University, Shizishan street No.1, Wuhan 430070, China; Huangyan District Fruit Tree Technology Promotion Station, Huanchengdong street No.258, Taizhou 318020, China; The Citrus Research Institute of Zhejiang Province, Zhejiang Academy of Agriculture Sciences, Yushanping street No.1, Taizhou 318020, China; National Key laboratory for Germplasm Innovation & Utilization of Horticultural Crops, Huazhong Agricultural University, Shizishan street No.1, Wuhan 430070, China; National Key laboratory for Germplasm Innovation & Utilization of Horticultural Crops, Huazhong Agricultural University, Shizishan street No.1, Wuhan 430070, China; National Key laboratory for Germplasm Innovation & Utilization of Horticultural Crops, Huazhong Agricultural University, Shizishan street No.1, Wuhan 430070, China; Huangyan District Fruit Tree Technology Promotion Station, Huanchengdong street No.258, Taizhou 318020, China; National Key laboratory for Germplasm Innovation & Utilization of Horticultural Crops, Huazhong Agricultural University, Shizishan street No.1, Wuhan 430070, China

## Abstract

Satsuma mandarin (*Citrus reticulata* “Unshiu”) is a global cultivar with superior fruit characteristics and ranking among the top citrus cultivars in terms of production. It is also a key contributor to citrus breeding. However, the lack of high-quality genome makes the origin of Satsuma mandarin has long been a matter of debate. Here, we assembled a gap-free, high-quality genome of Satsuma mandarin. Meanwhile, we collected and sequenced 15 indigenous citrus varieties in Zhejiang Province, 12 Satsuma mandarins, 21 citrus hybrids related to Satsuma mandarin, 10 modern citrus varieties, and 7 other mandarins. Through high-resolution genome analysis, we inferred that Satsuma mandarin originated from a cross between *C. reticulata* “Ruju” × *C. reticulata* “Bendiguang” and proposed that Satsuma mandarin most probably originated in East area in Zhejiang Province of China, where the two parents-like cultivars are still found in a sympatric region to date. These results provide new insights into the origin model of Satsuma mandarin. The spread of mandarin is also discussed, which probably associated with the culture exchange and trade activities between Japan and China from Tang Dynasty and afterwards.

## Introduction

Citrus is one of the most important fruit crops worldwide [1]. Citrus industry in China has long been dominated by mandarin and the mandarin production ranking first in China. China's citrus industry is dominated by mandarins, making it the world's largest producer, with over 50% of global citrus production (World Citrus Organization, 2021). Additionally, China is an important region of origin and domestication for citrus [2, 3].

Satsuma mandarin is a major type of citrus cultivar with superior fruit characteristics. It is the most widely cultivated mandarin variety worldwide [4] and the main variety commonly grown in eastern China [5]. There are over 300 varieties of Satsuma mandarin, known for their excellent quality, ease of cultivation, strong stress resistance, and wide adaptability.

East China is one of the most important commercialization site of mandarins [6]. Taizhou (Huangyan) and Wenzhou in Zhejiang Province have become key areas for mandarin cultivation [7] and are famous for many indigenous mandarin varieties [5, 6, 8] such as “Ruju” mandarin, “Bendiguang” mandarin, “Manju” mandarin, “Zaoju” mandarin, as well as recent cultivars like “Bendizao” mandarin, “Mantouhong” mandarin, “Hongyugan” mandarin, and “Kaixuangan” mandarin. Huangyan and Wenzhou have a long history of mandarin cultivation dating back to the Three Kingdoms period. The earliest historical records can be traced to the *Records of Terrestrial and Aquatic Animals in Linha* (264–280 AD), which mentions a citrus plant called “Jijuzi,” indicating that citrus cultivation in Huangyan dates back more than 1700 years.

Additionally, Satsuma mandarin plays a crucial role in modern citrus breeding. Hybrids with Satsuma mandarin as a parent can produce superior new varieties that are seedless, easy to peel, flavorful, and fragrant. By using Satsuma mandarin and its hybrids as parents, many notable varieties such as Kiyomi [9], Harehime [10], and Kara [11] have been developed. Currently, there are more than 100 hybrids of Satsuma mandarin, which are highly sought after and have significantly contributed to the development of the global citrus industry.

Origin research is particularly important for the domestication and improvement of perennial crops. While the origin and spread of some citrus resources have been reported [2, 12–15], the origin and spread of Satsuma mandarin, despite its significant role in modern citrus breeding, remain controversial. Around 1960, there was controversy over whether it originated in China [16] or Japan [17]. Subsequent studies have increasingly suggested that the Satsuma mandarin originated in China and later spread to Japan [18]. However, its exact origin in China is still debated [19]. In the past, the lack of high-quality genome and sequencing data prevented comprehensive analysis of the origin of Satsuma mandarin at the whole genome level. In 1963, the Zhejiang Citrus Research Institute suggested that “Bendiguang” mandarin might be the original species of Satsuma mandarin. In 1995, Fang and his colleagues conducted a study using polyacrylamide gel electrophoresis, which revealed that the isozymes of four Satsuma mandarin strains were completely consistent with those of the “Bendiguang” mandarin form the Huangyan region [20]. Hu used RAPD technology demonstrate a close relationship between “Bendiguang” mandarin and Satsuma mandarin [21]. Another study using DNA markers revealed that Satsuma mandarin is a hybrid between Kishu mandarin (*C. kinokuni* hort. ex Tanaka) and King mandarin (*C. nobilis* Lour. var. Kunep Tanaka) [22]. It is worth mentioning that Kishu mandarin is believed to have been introduced to Japan by “Nanfeng” mandarins from China [23].

Most Satsuma mandarin cultivars worldwide are derived from somatic mutations [24], resulting in a monogenetic background. Elucidating its origin will help improve the species and develop more diverse Satsuma mandarin varieties, better adapt to future climate change. A reference genome is essential for genetic studies, and thus acquiring a high-quality genome has persistently been pursued. The previously published Satsuma mandarin genome was based on illumina sequencing technology [25]. However, this version still contained thousands of gaps and lacked information on repetitive regions, centromeres, and telomeres, limiting access to variants in these regions. Fortunately, with advancements in sequencing technologies and computational algorithms, genome assembly has now progressed to telomere-to-telomere (T2T) sequencing [26]. This approach uses third-generation sequencing platforms, including PacBio high-fidelity long reads (HiFi), ultra-long reads from Oxford Nanopore Technologies (ONT), and Hi-C data [27, 28]. We assembled a gapless diploid genome of Satsuma mandarin and sequenced 65 *Citrus* resources. This data will help us understand the origin of Satsuma mandarin based on whole-genome sequencing.

## Results

### Phenotypic variation of Satsuma mandarin and related *citrus* species

**Figure 1:**
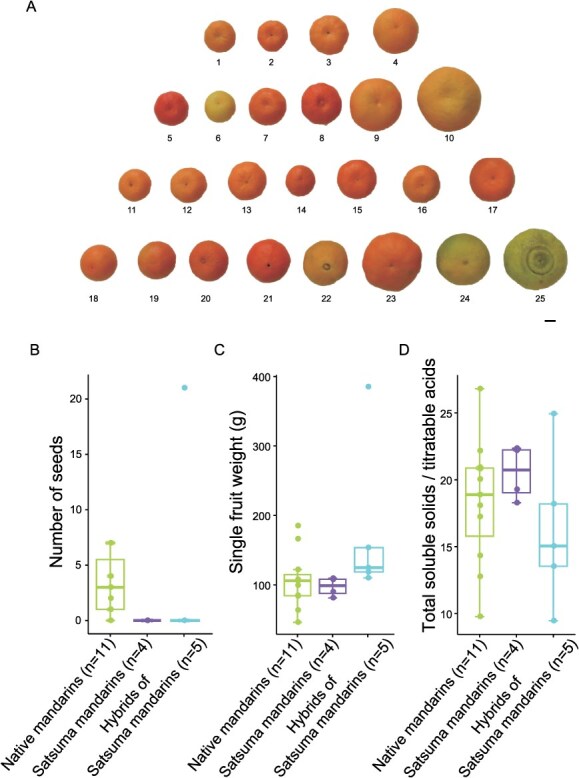
Phenotypic variation of Satsuma mandarins and related *Citrus* species. (A) Fruit appearance variation of Satsuma mandarins and related *Citrus* species. 1, “Nanfeng” mandarin; 2, “Ruju” mandarin; 3, “Zaoju” mandarin; 4, “Bendiguang” mandarin; 5, “Mantouhong” mandarin; 6, “Goutoucheng”; 7, “Manju” mandarin; 8, “Zhuhong” mandarin; 9, “Zhuluan” sour orange; 10, “Wenlinggaocheng”; 11, “Xinben No.1” mandarin; 12, “Bendizao” mandarin; 13, “Dongjiangbendizao” mandarin; 14, Yura; 15, Miyagawa wase; 16, Owari; 17, Oita; 18, “Hongshigan”; 19, Amakusa; 20, “Hongmeiren”; 21, Nankou Tangor; 22, Shiranui; 23, Kanpei; 24, Sweet spring; 25, “Chunxian”. Scale bar, 2 cm. (B-D) Number of seeds (B), single fruit weight (C) and total soluble solids/titratable acids (D) of Satsuma mandarins and related *Citrus* species in Zhejiang Province. Each box represents the median and interquartile range. The error bars represent standard error of the mean.

In tracing the parentage of over 70 hybrid cultivars, it was found that 23 of these cultivars are closely related to Satsuma mandarin (Supplementary Fig. 1). To elucidate the origin of Satsuma mandarin (*Citrus reticulata* “Unshiu”), we collected and sequenced a total of 65 *Citrus* accessions including 15 indigenous citrus varieties from Zhejiang Province, 12 Satsuma mandarins (some of which were bred in Japan), 21 citrus hybrids related to Satsuma mandarin, 10 modern citrus varieties and 7 other mandarin cultivars. These accessions varied in fruit size, flesh and peel color, degree of seedlessness, and fruit acidity (Fig. 1A-D, Supplementary Figs 2 and 3), suggesting a high level of genetic diversity among the *Citrus* species in this region.

### Gap-free genome assembly of Satsuma mandarin

A total of 32.93 Gb of PacBio HiFi long reads (with an N_50_ read length of ~14.48 kb and 102.91 × coverage) were generated to *de novo* assemble the genome of Satsuma mandarin (Supplementary Table 1). A 95.53 × Hi-C data were used to scaffold the assembled contig into pseudochromosomes (Supplementary Table 2 and Supplementary Fig. 4). We then used MUMmer [29] and the SWO.v3.0 genome (http://citrus.hzau.edu.cn/data/Genome_info/SWO.v3.0/SWO.v3.0.genome.fa) to order the 9 chromosomes (Supplementary Fig. 5). Our assembly had no gaps along all chromosomes, and the contig N_50_ reached to 34.11 Mb (Table 1). Compared with the previous version of Satsuma mandarin genome, substantial improvements in several metrics were observed in our assembly. The completeness of our genome assembly, as calculated by its Benchmarking Universal Single-Copy Orthologs (BUSCO) score, reached 98.30% for complete and single-copy orthologs. The contig N_50_ length was ~395-fold higher than that of the published Satsuma mandarin genome (386.40 kb) [25], and all gaps in the previously published genome were filled in the newly assembled genome.

**Figure 2:**
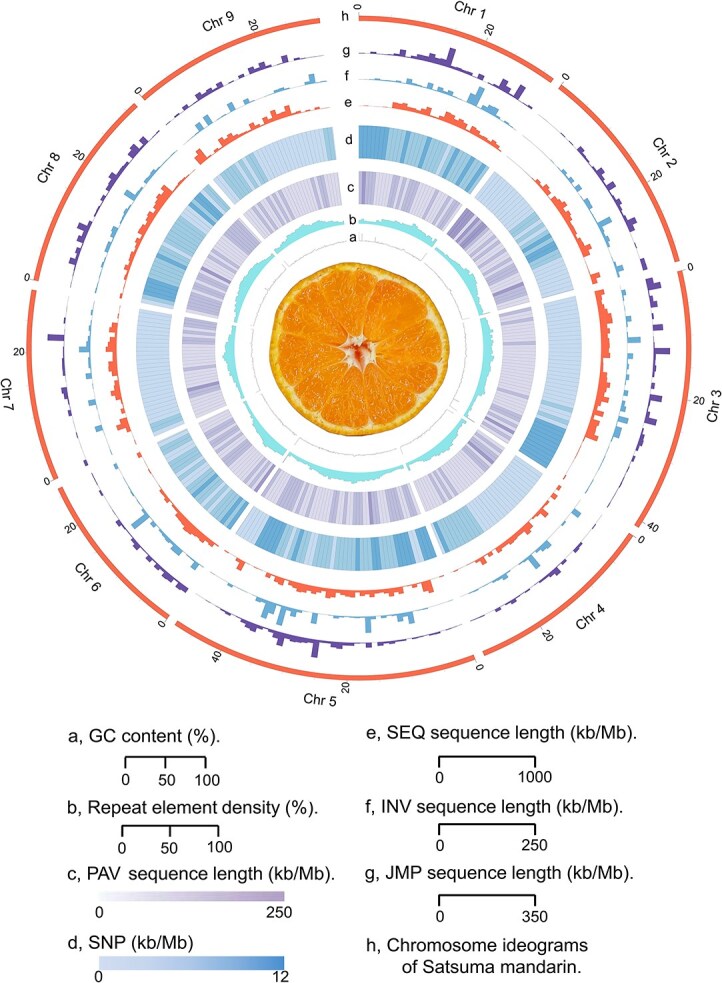
Characteristics of Satsuma mandarin genome. The fruit cross-section picture of Satsuma mandarin is at the center of the circle. (a) GC content (%), ranging from 0 to 100. (b) Repeat element density (%), ranging from 0 to 100. (c) PAV sequence length (kb/Mb), ranging from 0 to 250. (d) SNP number (× 1 000 SNPs, bin = 1 Mb), ranging from 0 to 12. (e) SEQ sequence length (kb/Mb), ranging from 0 to 1 000. SEQ represents a translocation event that requires jumping to a new query sequence in order to continue aligning to the reference. (f) INV sequence length (kb/Mb), ranging from 0 to 250. INV represents the beginning of the inverted region for a relocation event. (g) JMP sequence length (kb/Mb), ranging from 0 to 350. JMP represents the end of the inverted region for a relocation event. (h) Chromosome ideograms of the Satsuma mandarin genome (Mb scale)

The latest version of the Satsuma mandarin genome shows a significant improvement, with nearly 10% more transposable elements (TEs) newly assembled. We annotated 52.10% TEs in the newly assembled genome, compared to 42.47% in the published Satsuma genome. Among these, the LTR-Gypsy family exhibited the greatest enhancement in assembly completeness, increasing from 8.88% to 10.20% (Supplementary Tables 3 and 4). Additionally, we identified 14 277 presence and absence variations (PAVs), 1 599 794 single nucleotide polymorphisms (SNPs), 1 797 inversions, and 6 269 translocations between the two genomes (Supplementary Table 5). We observed that the differences between these genomes were particularly pronounced on chromosomes 1, 3, 5, and 8 (Fig. 2).

### Genetic analysis for Satsuma mandarin and related *citrus* species

**Figure 3:**
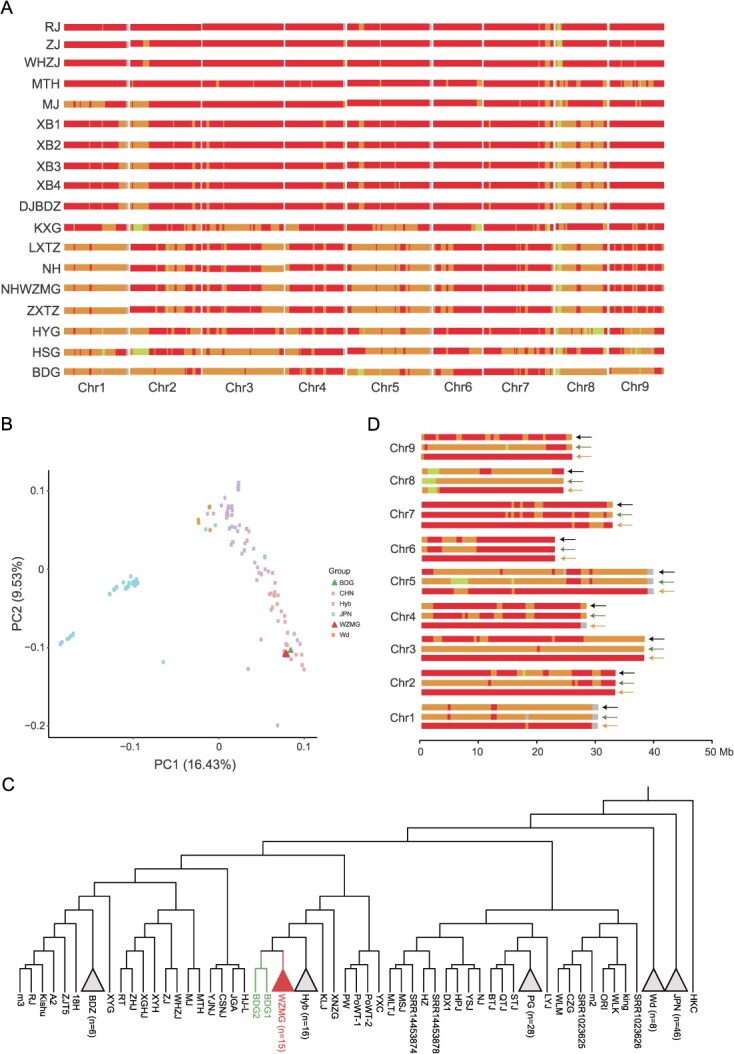
Genetic analysis of Satsuma mandarin and related *Citrus* species. (A) Genomic pattern of mandarins in Zhejiang Province. Red, homozygous segments with a mandarin origin; yellowish green, homozygous segments with a pummelo origin; orange, heterozygous segments with a mandarin/pummelo origin; gray, unknown region. The numbers of these resources are corresponding to Supplementary Table 6. (B) PCA of 11 Satsuma mandarins (WZMG), 8 wild mandarins (Wd), 55 mandarins from Japan (JPN), 37 citrus hybrids (Hyb), 71 mandarins from China (CHN), and 2 “Bendiguang” mandarins (BDG). (C) A phylogenetic tree constructed using SNP data. The codes of these resources correspond to Supplementary Table 6. (D) Genomic landscape of Satsuma mandarin (black arrow), “Bendiguang” mandarin (green arrow), and “Ruju” mandarin (orange arrow)

We newly sequenced 65 Satsuma mandarins and related *Citrus* species (Supplementary Table 6), along with 93 pummelos, 111 mandarins, 31 sweet oranges, 69 sour oranges, and 26 other related citrus resources previously published (Supplementary Table 7). The genomic landscape analysis revealed native populations of pure mandarins in Zhejiang Province, such as “Ruju” mandarin (RJ), “Zaoju” mandarin (ZJ), “Wuhe Zaoju” mandarin (WHZJ), and “Mantouhong” mandarin (MTH). There are also domesticated mandarins, such as “Bendiguang” mandarin (BDG) and “Bendizao” mandarin (XB1–4, DJBDZ) (Fig. 3A and Supplementary Fig. 6). Both principal component analysis (PCA) and phylogenetic analyses divided mandarins from Japan (new species of mandarin citrus) [30] and China into two distinct groups (Fig. 3B and C). Notably, all Satsuma mandarins coalesced into a single point on the PCA plot, closer to the native mandarins collected from Zhejiang Province than to other mandarins (Fig. 3B and C). This suggests that mandarins from the Zhejiang Province may be closely related to Satsuma mandarins. We identified the “Bendiguang” mandarin from Zhejiang Province, which is similar to but not identical to the Satsuma mandarin (Fig. 3D). These results indicate an abundance of Satsuma mandarin-like accessions in Zhejiang Province. We confirmed that Satsuma mandarins share a nearly identical genomic landscape (Fig. 3A), consistent with the fact that they arise from somatic mutations [24, 31].

### Elucidating the origin of Satsuma mandarin

**Figure 4:**
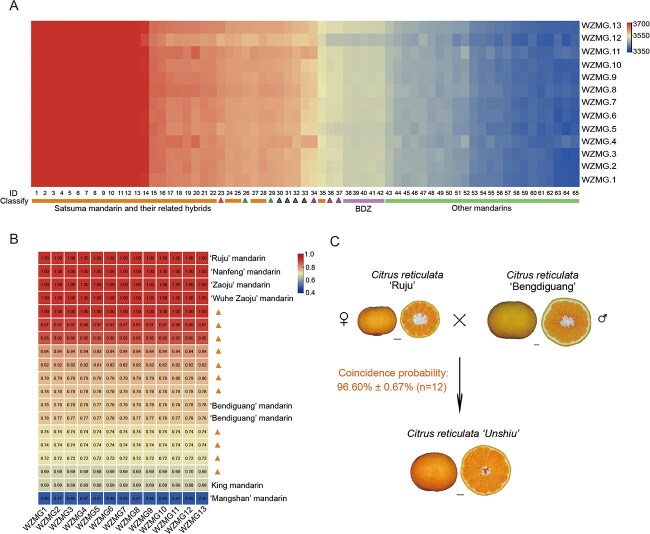
Origin of Satsuma mandarin. (A) Kinship matrix between Satsuma mandarins and related *Citrus* species. Each row of the heatmap represents a recipient copying vector showing the number of fragments shared between the recipient and each donor (columns), with block-like patterns are observed on the heatmap. Blue represents less haplotype sharing, and red indicates more haplotype sharing. BDZ, “Bendizao” mandarin. Red triangles, “Ruju” mandarin. Red triangle with black border, Kishu mandarin. Red triangle with purple border (varieties of “Ruju” mandarin, including 34, “Nanfeng” mandarin from Jiangxi Province; 36–37, “Zaoju” mandarin and “Wuhe Zaoju” mandarin from Zhejiang Province). Green triangles, “Bendiguang” mandarins. Green triangles with black border, Kunenbo mandarins. The numbers of these resources correspond to Supplementary Table 8. (B) IBD analysis among Satsuma mandarins and related *Citrus* species. Orange triangles, hybrids of Satsuma mandarin. Red indicates a close relationship. The numbers in the heatmap represent the values of IBD1. (C) Model for the origin of Satsuma mandarin. The coincidence probability refers to the probability that a genome landscape matches the genetic relationships between the parents and hybrids

We used whole-genome resequencing data from 389 *Citrus* species to assess their relatedness to Satsuma mandarin. The kinship matrix suggested that “Ruju” mandarin and “Bendiguang” mandarin are most closely associated with Satsuma mandarin, aside from Satsuma mandarin-derived cultivars (Fig. 4A, Supplementary Tables 8 and 9). In addition, both “Ruju” mandarin and “Bendiguang” mandarin are distributed in Zhejiang Province. Identity by descent (IBD) analyses based on whole-genome SNP data indicated that “Ruju” mandarin, “Bendiguang” mandarin, and Satsuma mandarin-derived cultivars are more closely related to Satsuma mandarin than other mandarins. The probability that “Ruju” mandarin and Satsuma mandarin share a set of chromosomes is 100%, consistent with a parental relationship between “Ruju” mandarin and Satsuma mandarin (Fig. 4B). An analysis of the chloroplast genomes of the *Citrus* species revealed that “Ruju” mandarin is closer to Satsuma mandarin than “Bendiguang” mandarin, suggesting that “Ruju” mandarin is more likely the seed parent of Satsuma mandarin (Supplementary Fig. 7).

**Table 1:** Gap-free genome statistics for Satsuma mandarin

Feature	*Citrus reticulata* “Unshiu”
Assembled size (Mb)	346.63
BUSCO completeness of assembly (%)	98.30
Number of gaps	0
Contig N_50_ (Mb)	34.11
Percentage of transposable elements (%)	54.41
Number of gene models	27 580

A recent study proposed that the Satsuma mandarin originated from a cross between Kishu mandarin as the seed parent and Kunenbo mandarin as the pollen parent [22]. Our results indicated that “Ruju” mandarin has a higher chance than Kishu mandarin, while “Bendiguang” mandarin has a similar probability to Kunenbo mandarin of being the parent of Satsuma mandarin (Fig. 4A and Supplementary Tables 8 and 9). Specifically, for the seed parent of Satsuma mandarin, we found that the average number of shared fragments between “Ruju” mandarin (RJ), Kishu mandarin (SRR6188456), “Nanfeng” mandarin (m3), “Zaoju” mandairn (ZJ), “Wuhe Zaoju” mandarin (WHZJ), and Satsuma mandarin was 3 610.75, 3 569.17, 3 565.55, 3 520.91, and 3 520.76, respectively (a higher number indicates closer association with Satsuma mandarin). For the pollen parent of Satsuma mandarin, the average number of shared fragments between “Bendiguang” mandarin (BDG), Kunenbo mandarin (SRR14453877), and Satsuma mandarin was 3 597.62 and 3 584.35, respectively.

With the extant germplasm of both parents of Satsuma mandarin in the Huangyan region of Zhejiang Province and the above genomic inference, we propose that Satsuma mandarin originated from a cross between “Ruju” mandarin as the seed parent and “Bendiguang” mandarin as the pollen parent. We analyzed the SNPs that matched the hybridization model based on data from 12 030 766 SNPs, and calculated the probability of Satsuma mandarin being derived from “Ruju” mandarin and “Bendiguang” mandarin to be 96.60% ± 0.67% (Fig. 4C and Supplementary Table 10). In addition, the genome landscape (calculated using 1 014 134 species-specific SNPs) also supports this inference (Fig. 3D and Supplementary Fig. 6 and and 8).

## Discussion

A complete reference genome is crucial for both genetic origin research and molecular breeding [32, 33]. A gap-free genome was assembled for Satsuma mandarin. Based on whole genome research, we have identified that “Ruju” mandarin is the seed parent of Satsuma mandarin and “Bendiguang” mandarin serves as its pollen parent. Notably, the Huangyan area is the only known region in China where both parental lines coexisted, providing a high chance for natural hybridization between them. In a recent study, Tokurou and his colleagues proposed that Satsuma mandarin originated from a cross between Kishu mandarin (*C. kinokuni* hort. ex Tanaka) as the seed parent and Kunenbo mandarin (*C. nobilis* Lour. var. kunep Tanaka) as the pollen parent. The authors mentioned that Kishu mandarin is identical to “Nanfeng” mandarin, a very old mandarin with a long history of cultivation in Nanfeng County, Jiangxi Province, China [25]. In our genomic analysis, we confirmed that Kishu mandarin is identical to “Nanfeng” mandarin in Jiangxi Province (Supplementary Fig. 6 and 8). Through whole-genome high-resolution analysis, our results indicated that “Ruju” mandarin has a higher chance than “Nanfeng” mandarin of being the seed parent of Satsuma mandarin (Fig. 4A). The origin of Kishu mandarin is not clearly known, but it is speculated to have been transmitted from China to Japan in ancient times [34–38]. For the pollen parent, Tokurou and his colleagues mentioned that one group of King mandarin (*C. nobilis* Lour. var. Kunep Tanaka), named Kunenbo-A, is identical to “Bendiguang” mandarin, which is also inferred to be pollen parent in our model. Moreover, our result showed that “Bendiguang” mandarin has a similar chance as Kunenbo mandarin of being the pollen parent of Satsuma mandarin (Fig. 4A). Notably, the extant germplasm of both parents of Satsuma mandarin in the Huangyan region of China and Kunenbo mandarin (a type of King mandarin) is not an indigenous variety of Japan but is regarded as having been transmitted from South China [37–39]. Thus, we propose that “Ruju” mandarin and “Bendiguang” mandarin were more likely the parents of Satsuma mandarin. Moreover, we can construct a hybridization model based on the inferred parents of Satsuma mandarin to introduce diversity and break the monogenetic background of Satsuma mandarin.

Satsuma mandarin is a high-quality cultivar characterized by being seedless, low in acid and high in sugar. A recent study showed that miR399-CsUBC24 regulates male sterility of Satsuma mandarin [40], and another study identified a 920-kb genomic region associated with seedlessness, spanning from 6 184 904 to 7 104 658 bp on scaffold 8 of the *C. clementina* genome v1.0 [41]. We used Mummer software [29] to identify the homologous segment in the sweet orange genome, which includes 76 genes (Supplementary Table 11). Further variation analysis revealed variants in the genic regions or promoter regions of four genes that were highly linked with seedlessness of Satsuma mandarin (Supplementary Fig. 9). Additionally, we identified lowly expressed transporter and proton-pumping genes of Satsuma mandarin, such as PH1 (Cs1g_pb020070) and PH5 (Cs1g_pb015530). Furthermore, we identified the sugar transport gene SWEET2 (Cs5g_pb016540), which was highly expressed of Satsuma mandarin (Supplementary Table 12).

The origin place of Satsuma mandarin has been a long controversy. Based on our research and historical evidence, we infer that Satsuma mandarin more likely originated in East China rather than Japan. According to the Japanese Citrus Center, the name “Wenzhou mandarin orange” is derived from Wenzhou Prefecture in China, which has been renowned for its mandarin production since the Tang and Song dynasties. During the Southern Song Dynasty, Han Yanzhi wrote the world's first monograph on citrus, “*Ju Lu*,” which systematically documented the citrus varieties and cultivation techniques in the Wenzhou area. These records suggest that East China, including the ancient Huangyan or Wenzhou region, is most likely the origin place of Satsuma mandarin.

The spread of mandarins is closely associated with cultural exchanges and trade activities between China and Japan. Taizhou, especially Huangyan, became an important citrus production area after the Tang Dynasty (618–907 A.D.) and a key hub for maritime trade between China and Japan. Additionally, the Tiantai Guoqing Temple and Huangyan Ruiyan Temple were highly esteemed during the Tang Dynasty. It is possible that citrus from Taizhou, especially Huangyan, attracted attention and was introduced to Japan. Coincidentally, Japan's “*Kojiki*” records state that mandarins were introduced to Japan from China during the Tang Dynasty in 712. According to the “*History of the Song Dynasty*” Japanese monks in the Northern Song Dynasty (960–1127 A.D.) frequently traveled between China and Japan through Mingzhou (now Ningbo, near Huangyan). Taizhou, especially Huangyan, had ancestral temples and thus held an extremely important position in the history of Sino-Japanese maritime trade and Buddhist cultural exchanges. Particularly in the Southern Song Dynasty, coinciding with Japan's introduction of Tiantai tea species, citrus from Taizhou was also considered a specialty. It is possible that citrus from Taizhou was introduced to Japan during this period. Coincidentally, Japanese sources such as “*Citrus”*, “*Japanese Garden Dictionary”*, and “*Japanese Garden Chronicle”* by Ikuro Takahashi suggest that Japan's Kishu mandarin originated from the Yangtze River region in China and was introduced from southern China during the Song and Yuan Dynasties, approximately 700–800 years ago. According to Japanese scholar Ikuro Takahashi, over 600 years ago, during the Ming Dynasty in China, a Japanese monk named “Zhihui” came to study at the Guoqing Temple on Tiantai Mountain in Zhejiang Province. He passed through Huangyan and brought mandarins back to Kyushu, planting the seeds on Ozhong Island (now Kagoshima), which eventually led to the selection of a seedless variety. Tanaka proposed that Satsuma arose in Nagashima town in Kagoshima Prefecture between the 15th and 16th centuries [37, 38]. Around the second year of Yongle (1404), China and Japan exchanged goods at a designated port (Ningbo or Wenzhou, near Huangyan) under the guise of tribute. Therefore, it is plausible that citrus varieties from Huangyan were introduced and cultivated in Kagoshima. In 1709, Kaibara described 15 citrus varieties, including “Unshukitsu”, an old name for Satsuma [39].

## Materials and methods

### Plant materials and whole-genome sequencing

A total of 65 mandarins were sequenced in this study (Supplementary Table 6). At least 10 μg of genomic DNA from each accession was used to construct the sequencing libraries. Paired-end sequencing libraries with an insert size of approximately 200–500 bp were constructed and sequenced on the Illumina HiSeq 2500 platform.

### Genome assembly and annotation

PacBio HiFi reads were generated using the PacBio Sequel II platform. The initial output of Hifiasm (v0.16.1) [42] yielded the draft assembly. We also generated Hi-C reads to anchor contigs using the 3d-dna pipeline (v180922). RepeatModeler2 (v1.0.11) [43] was first used to construct a transposable element (TE) library. The self-built TE libraries and the plant TE library in Repbase were then used to mask the genomes with RepeatMasker (v4.0.9) [44]. *Ab initio* gene predictions, homology searches, and transcriptome deep sequencing (RNA-seq) were integrated to annotate genes. AUGUSTUS (v.3.3) [45], SNAP [46], and GlimmerHMM (v3.0.4) [47] were used to perform *ab initio* gene predictions. GenomeThreader software [48] was used to perform homology searches. Hisat2 (v2.2.1) [49] and Cufflinks (v2.2.1) [50] were used for RNA-seq read alignment and transcript construction. Trinity (v.2.4.0) [51] was used for transcript assembly. The transcripts were aligned to genomes to predict genes using PASA (v2.4.1). The data from these steps were integrated by EVM (v1.1.1) [52]. The functions of the predicted genes were assigned using eggNOG-mapper [53].

### Comparative genomic analyses

LASTZ (v1.02.00) [54] and the nucmer program in MUMmer (v4.0.0) [29] were used for genomic sequence alignment. One-to-one alignments were then filtered using the delta-filter program in the MUMmer package, with a minimum alignment length of 20 000 bp (delta-filter -i 90 -l 20 000). SNPs and SVs were obtained using the show-diff and show-snps (-Clr -× 1) program in the MUMmer package.

### Genome mapping and variant calling

Raw Illumina reads were processed to remove adapter sequences and low-quality reads. The cleaned paired-end reads of all accessions were mapped to the reference genome (http://citrus.hzau.edu.cn/data/Genome_info/SWO.v2.0/SWO.v2.0.genome.fa) using BWA (v0.7.17) [55]. Duplicate mapping reads were removed using the MarkDuplicates command from GATK (v4.1.1) [56]. SNPs were called using HaplotypeCaller command from GATK (v4.1.1). The raw VCF file was filtered using VCFtools (v0.1.13) [57].

### PCA

To analyze the genetic relationships among all 389 *Citrus* species, we used all the SNPs called through the above process and performed PCA with default settings using GCTA (v1.26.0) [58]. We used PLINK (v1.90) [59] for format conversion and the R package *prcomp* to calculate PC1 and PC2.

### Evaluation of genetic relationship

We inferred the genetic relationship based on haplotype polymorphisms at the population level [60, 61]. Before calculating kinship, we first pruned the SNPs called in the previous section (*r*^2^ > 0.9) using PLINK (v1.90) and phased the filtered SNPs using Beagle 4.0 software (v r1399) [62]. We then used the ChromoPainter and FineSTRUCTURE pipeline [60] to analyze 364 citrus resources. The genetic relationship was constructed at the haplotype level, and a genetic relationship matrix was calculated to investigate the proportion of haplotypes each individual contributed to the remaining individuals. In addition, we also calculated IBD to assess the relatedness of different citrus accessions to Satsuma mandarin using PLINK (v1.90). A combination of customized R scripts, provided by the authors and developed by us, was used to visualize the results.

### Fruit quality determination of hybrids

Soluble solids (TSS) and titratable acid (TA) were determined using a PAL-BX/ACID sugar and acid integrated machine. The measurements were repeated three times. Single fruit weight was determined using an electronic balance with a sensitivity of 0.01 g. The weight of three fruits was measured to ensure biological repetition, and the average value was taken. The fruit shape index was calculated as the longitudinal diameter value divided by the transverse diameter value. A vernier caliper was used to measure the distance from the top of the fruit to the pedicle end as the longitudinal diameter value (mm), and the cross-sectional diameter of the fruit as the transverse meridian value (mm). Three fruits were measured from each fruit tree to ensure biological repetition, and the average value was taken.

## Supplementary Material

Web_Material_uhaf015

## Data Availability

Genome assemblies have been deposited in the National Genomics Data Center (https://ngdc.cncb.ac.cn/) under accession number GWHERCA00000000 (*C. reticulata* “Unshiu”). The raw data for the assembly have been deposited in the National Genomics Data Center (https://ngdc.cncb.ac.cn/) under accession PRJCA022822 and in NCBI under accession PRJNA1064391. The detailed accession numbers for the 65 whole-genome sequencing data are listed in Supplementary Table 6. The assembled genome and annotation are also available at http://citrus.hzau.edu.cn/.
