# Supplementary material for: Genomic origin of Citrus reticulata “Unshiu”
Source: Hortic Res. 2025 May 1;12(5):uhaf015. doi: 10.1093/hr/uhaf015 (PMC11966385; doi:10.1093/hr/uhaf015)
Supplement: Web_Material_uhaf015 [file web_material_uhaf015.zip › Supplementary Figure 2024.12.15.docx]

**Supplementary Figures**

**Genomic origin of *Citrus reticulata* ‘Unshiu’**


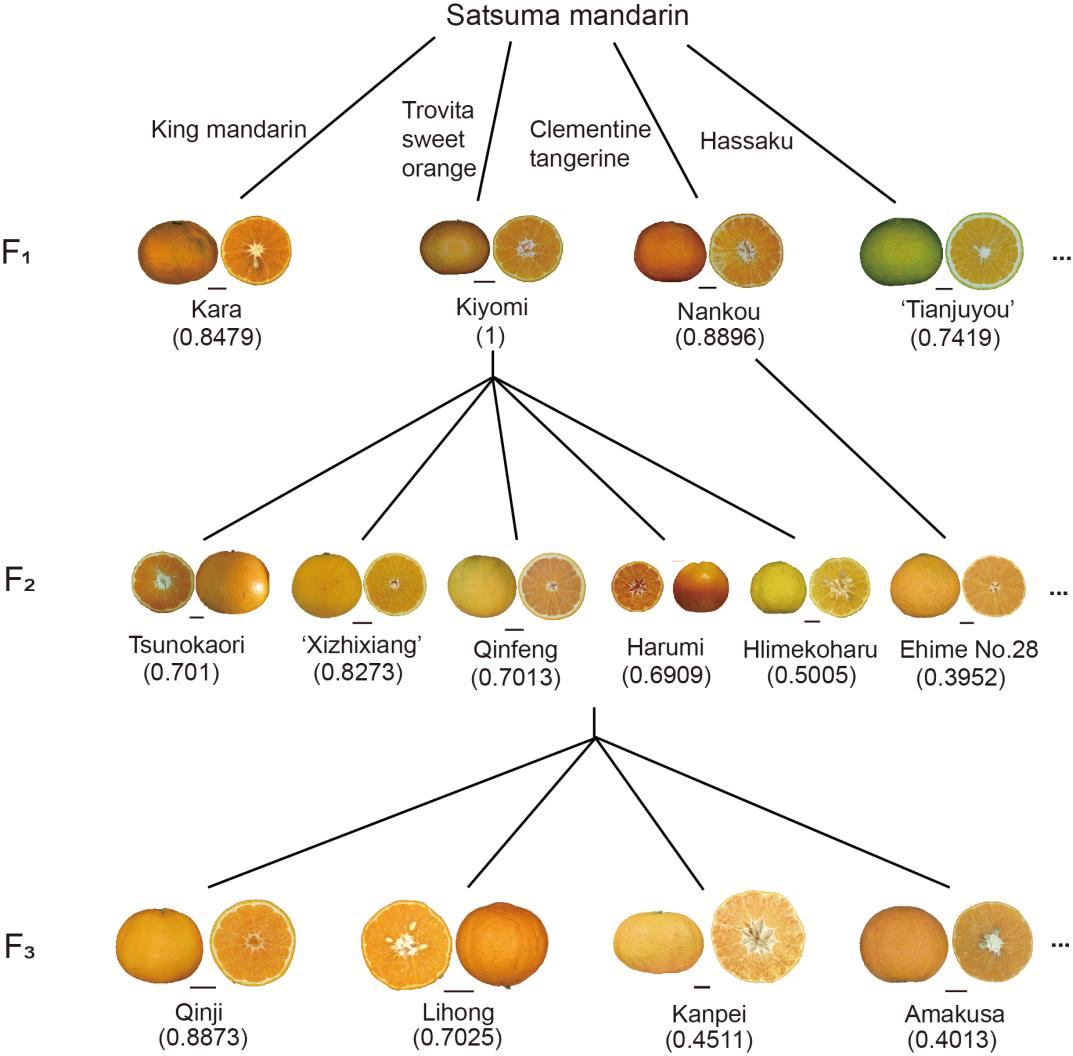


**Supplementary Fig. 1. The impact of Satsuma mandarin on modern citrus breeding.**

The numerical value represents the IBD1 shared between the resource and Satsuma mandarin.

**
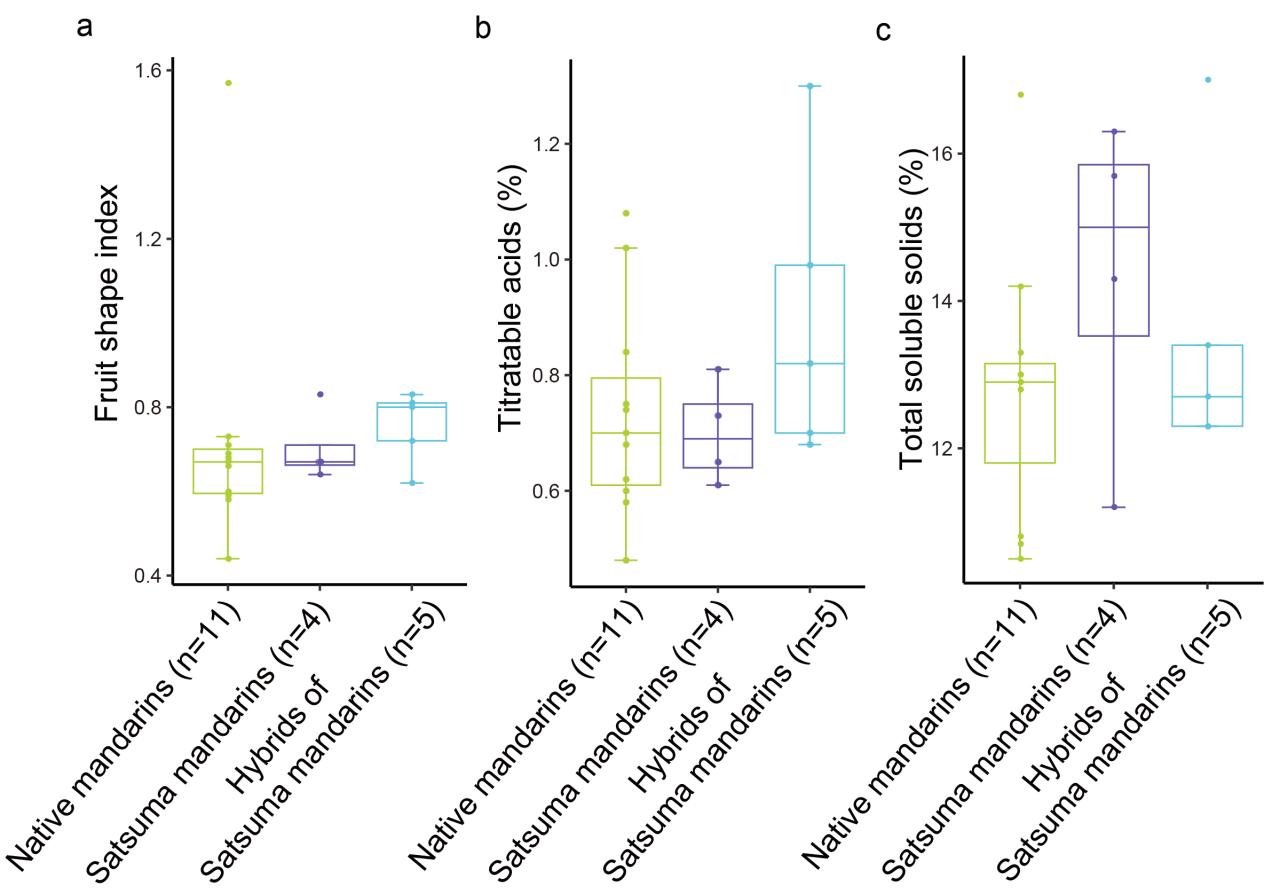
**

**Supplementary Fig. 2. Phenotypes of Satsuma mandarins and related *Citrus* species in Zhejiang Province.**

**(a)** Fruit shape index, **(b)** titratable acids, and **(c)** total soluble solids of Satsuma mandarins and related *Citrus* species. Each box represents the median and IQR. The error bars represent the standard error of the mean.

**
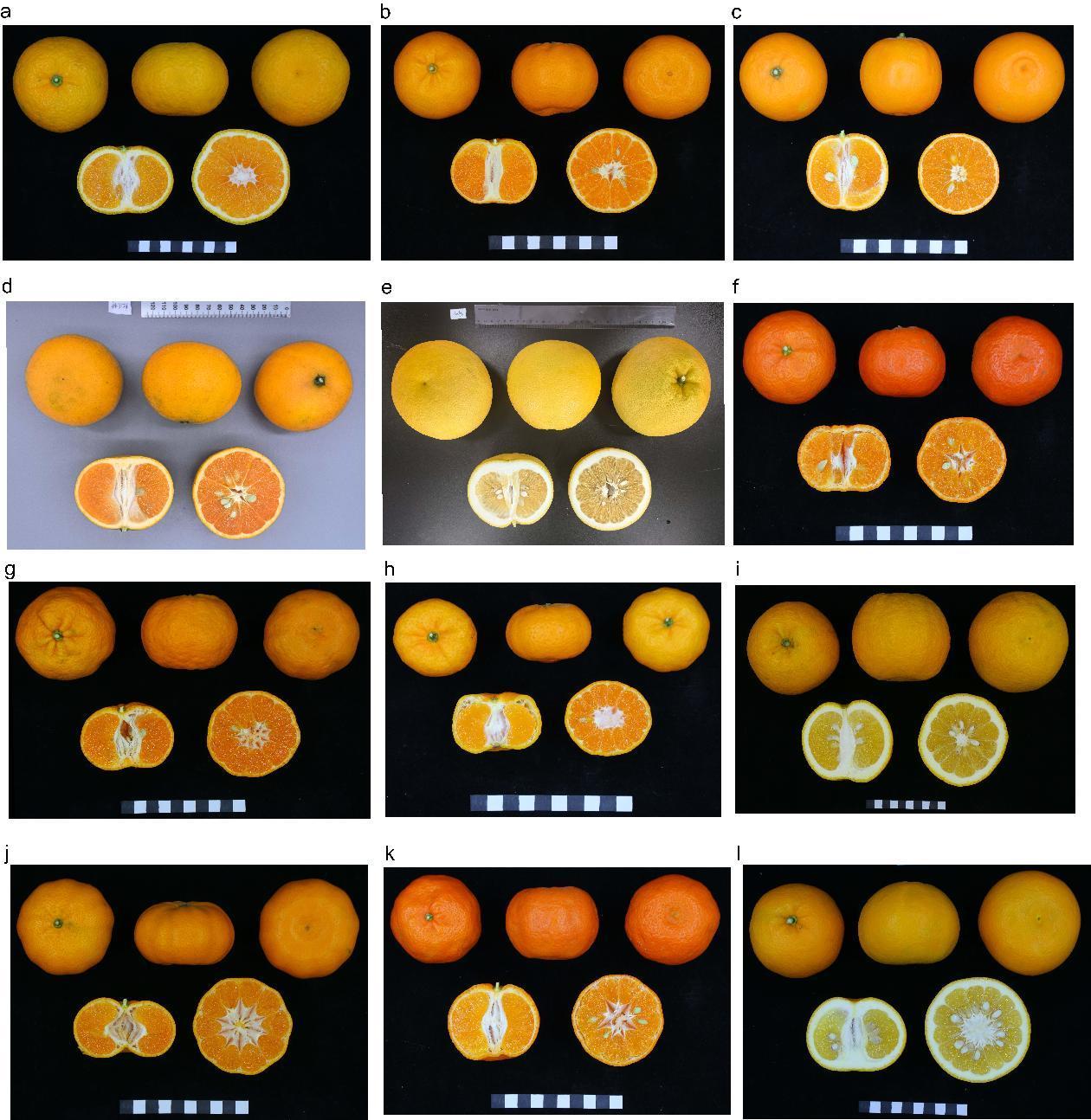
**

**Supplementary Fig. 3. Fruit shape of native mandarins and citrus hybrids in Zhejiang Province.**

**(a)** ‘Bendiguang’ mandarin, **(b)** ‘Bendizao’ mandarin, **(c)** ‘Hongshigan’ mandarin, **(d)** ‘Hongyugan’ mandarin, **(e)** ‘Huyou’, **(f)** ‘Mantouhong’ mandarin, **(g)** ‘Manju’ mandarin, **(h)** ‘Ruju’ mandarin, **(i)** ‘Wenlinggaocheng’, **(j)** ‘Zaoju’ mandarin, **(k)** ‘Zhuhong’ mandarin, **(l)** ‘Zhuluan’ sour orange. Scale bar, 1 cm.

**
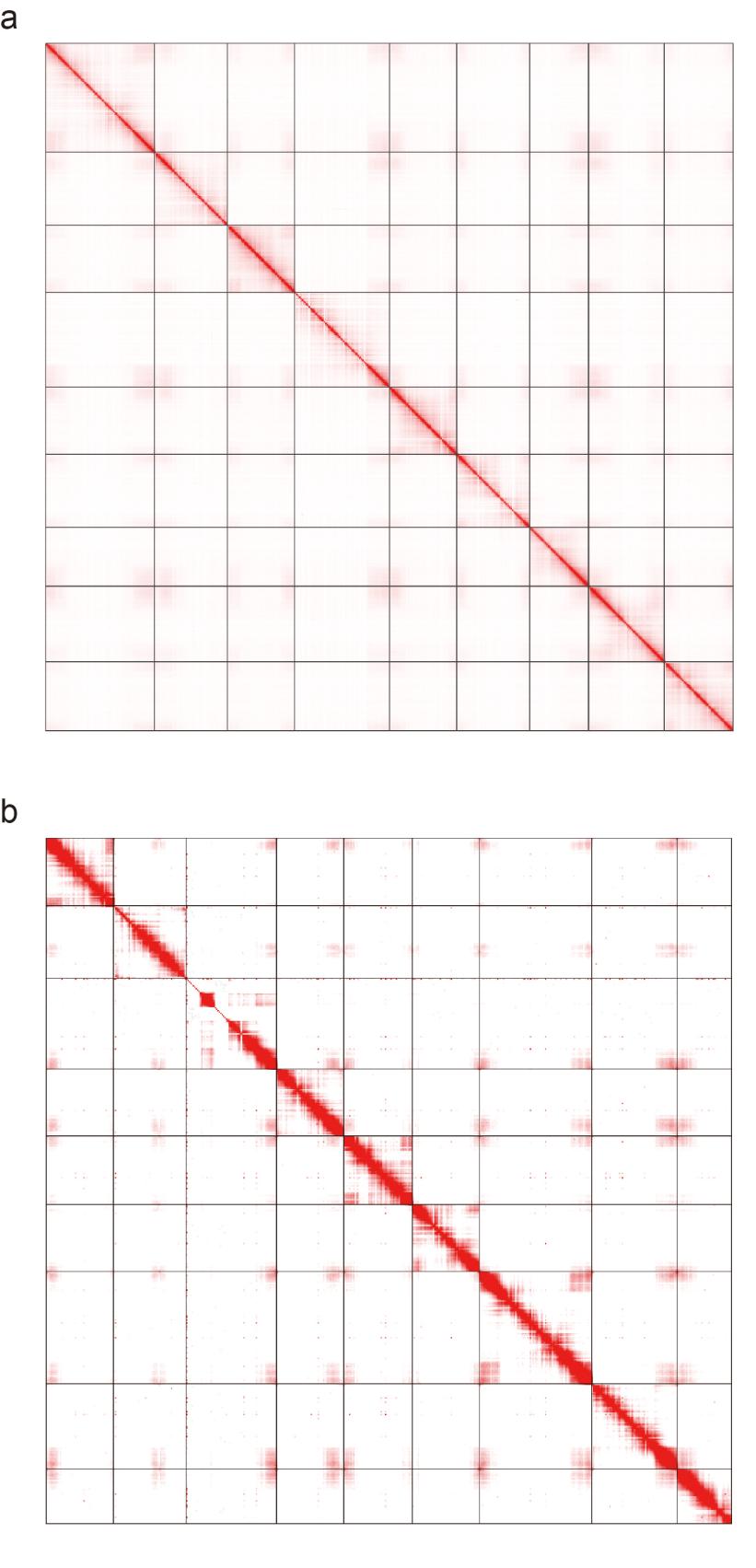
**

**Supplementary Fig. 4. Hi-C chromatin interaction map of *Citrus reticulata* ‘Unshiu’ genome assembly.**

Color represents the density of Hi-C interactions, red indicates larger number of links.

**
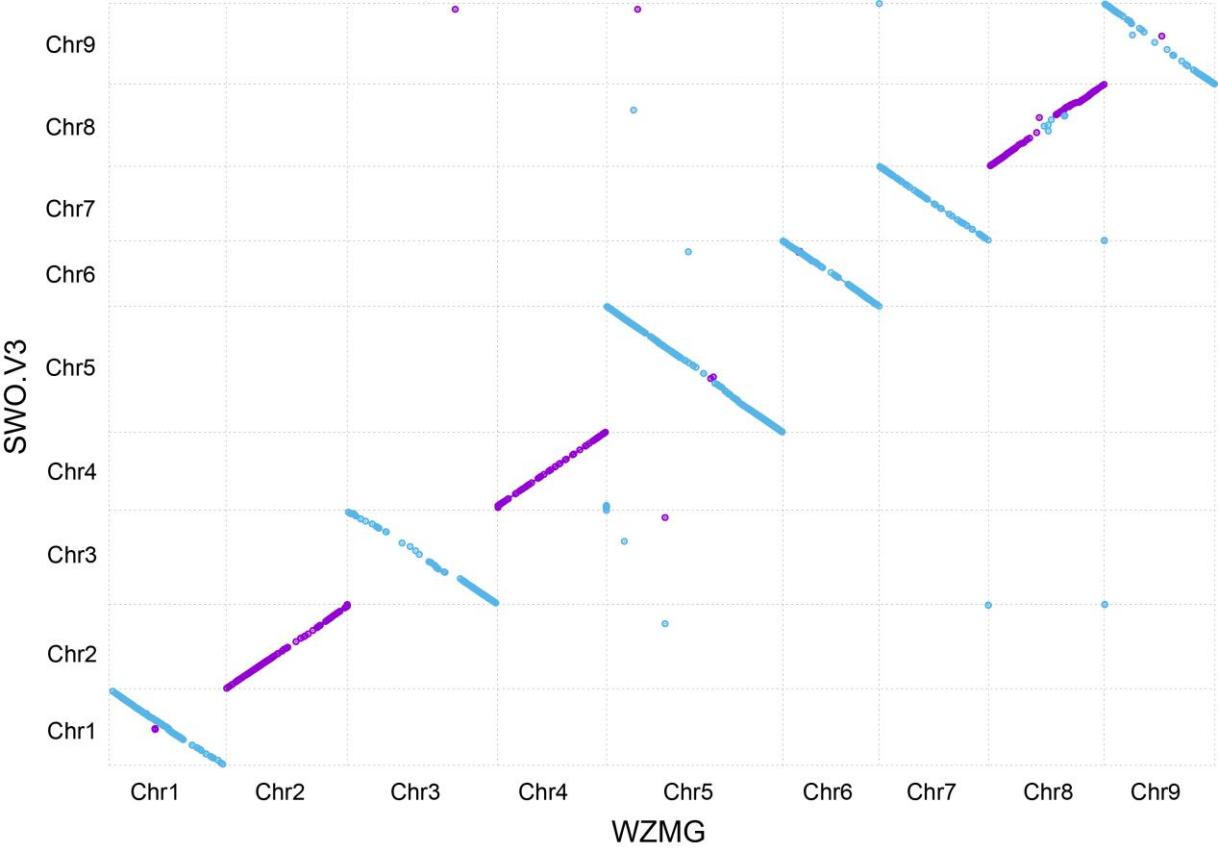
**

**Supplementary Fig. 5. Genomic synteny between *Citrus reticulata* ‘Unshiu’ and previously published haploid sweet orange genome.**

The genomic synteny between *Citrus reticulata* ‘Unshiu’ and SWO.V3 genome. SWO.V3 was download from <http://citrus.hzau.edu.cn/data/Genome_info/SWO.v3.0.>

**
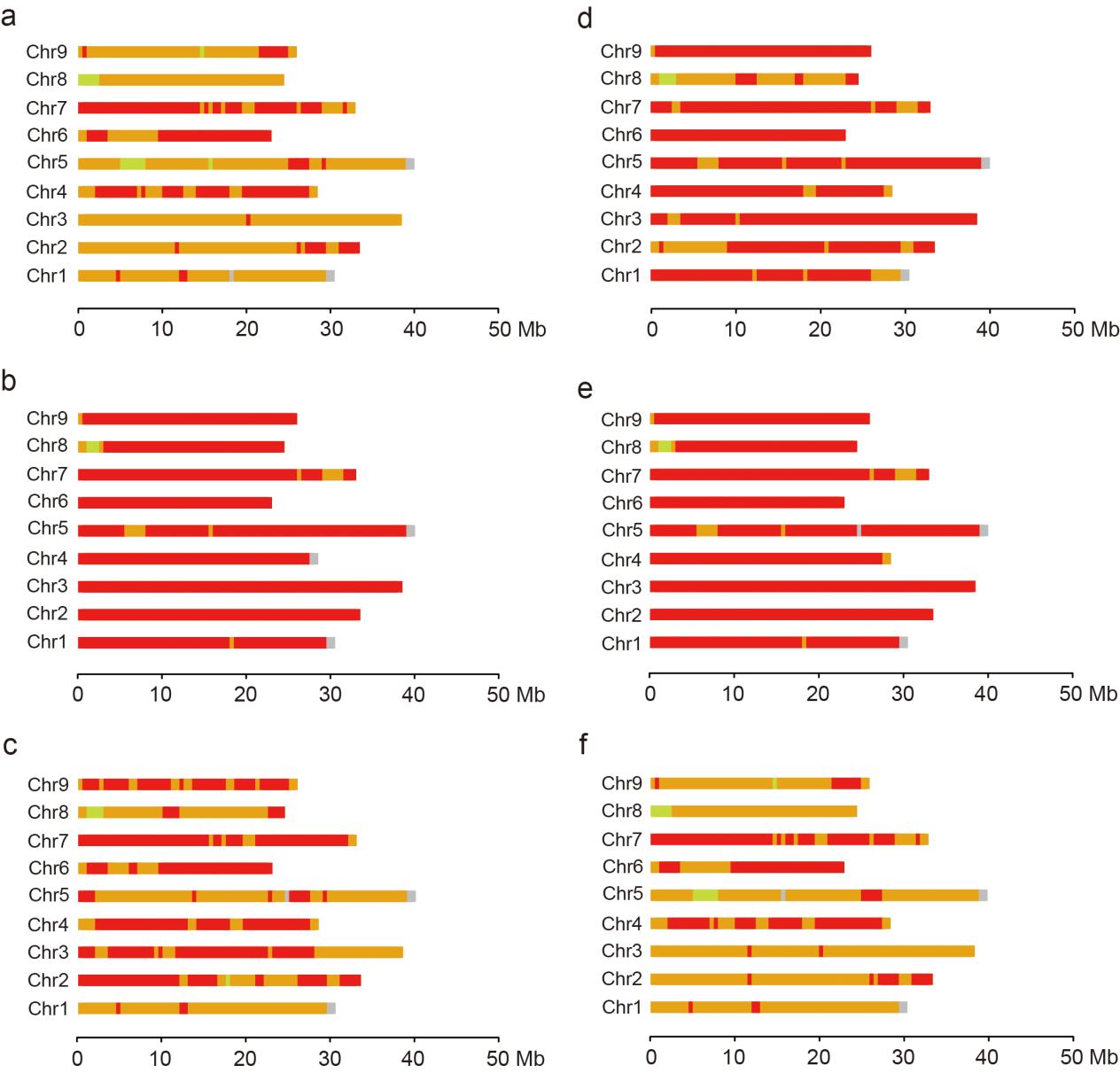
**

**Supplementary Fig. 6. Genomic landscape of ‘Ruju’ mandarin, ‘Bendiguang’ mandarin, Satsuma mandarin, ‘Bendizao’ mandarin, Kishu mandarin, and Kunenbo mandarin.**

Genomic patterns of ‘Bendiguang’ mandarin **(a)**, ‘Ruju’ mandarin **(b)**, Satsuma mandarin **(c)** , ‘Bendizao’ mandarin **(d)**, Kishu mandarin **(e)**, and Kunebo mandarin **(f)**. Red, homozygous segments with a mandarin origin; yellowish green, homozygous segments with a pummelo origin; orange, heterozygous segments with a mandarin/pummelo origin; gray, unknown region.

**
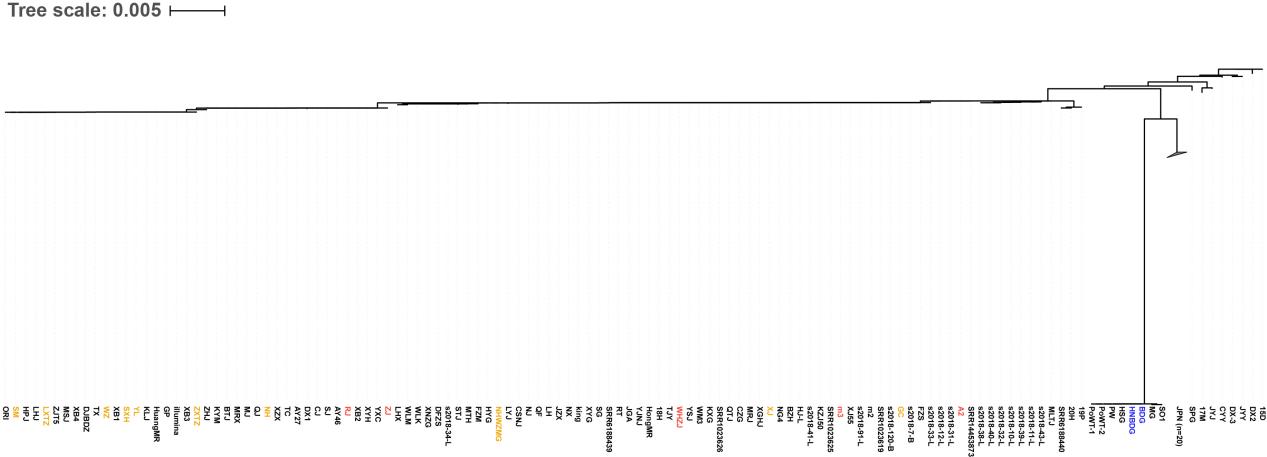
**

**Supplementary Fig. 7. Phylogeny analysis of chloroplast genome sequences of Satsuma mandarins and related *Citrus* species.**

A Maximum Likelihood tree was generated from SNPs between Satsuma mandarins and related *Citrus* species. Yellowish green, Satsuma mandarins. Red, ‘Ruju’ mandarin and its varieties. Blue, ‘Bendiguang’ mandarins.


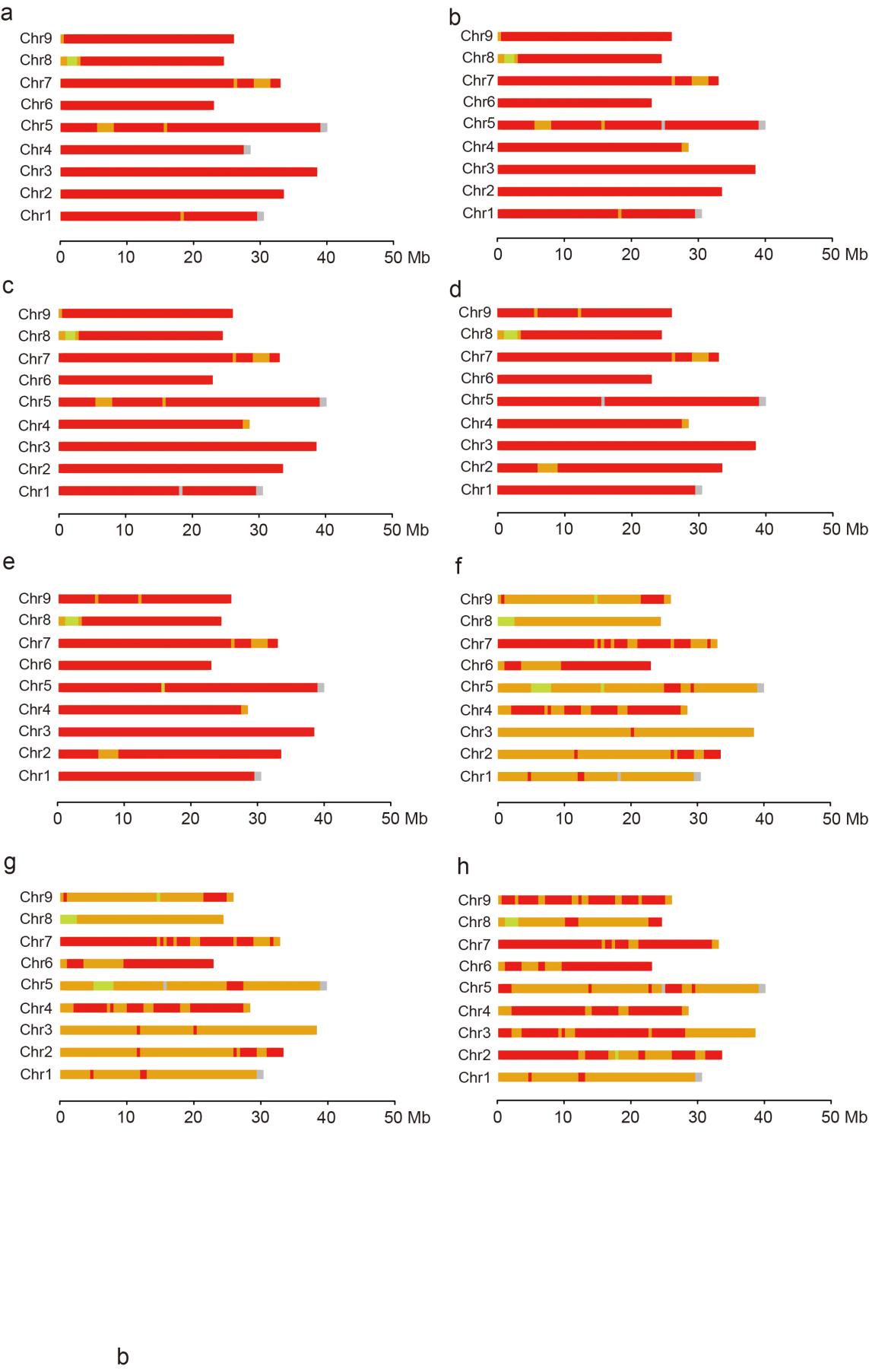


**Supplementary Fig. 8. Genomic landscape of ‘Ruju’ mandarin, Kishu mandarin, Nanfeng mandarin, ‘Zaoju’ mandarin, ‘Wuhe Zaoju’ mandarin, ‘Bendiguang’ mandarin, Kunebo mandarin and Satsuma mandarin.**

Genomic pattern of ‘Ruju’ mandarin **(a)**, Kishu mandarin **(b)**, Nanfeng mandarin **(c)**, ‘Zaoju’ mandarin **(d)**, ‘Wuhe Zaoju’ mandarin **(e)**, ‘Bendiguang’ mandarin **(f)**, Kunebo mandarin **(g)** and Satsuma mandarin **(h)**. Red, homozygous segments with a mandarin origin; yellowish green, homozygous segments with a pummelo origin; orange, heterozygous segments with a mandarin/pummelo origin; gray, unknown region.

**
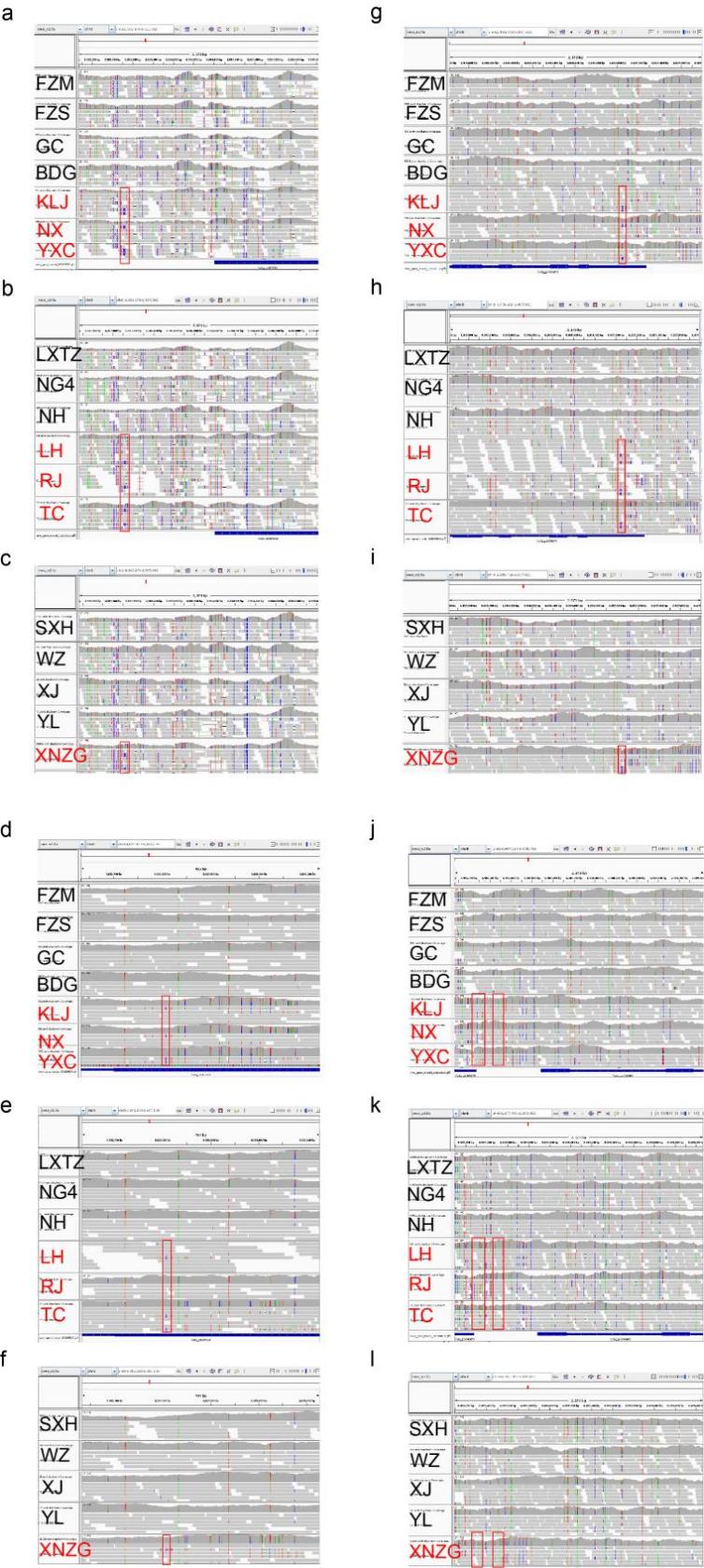
Supplementary Fig. 9. Variations in the genic or promoter regions of four genes that are highly linked with seedlessness of Satsuma mandarin.**

Variations around Cs8g_pb007380 **(a-c)**, Cs8g_pb007220 **(d-f)**, Cs8g_pb006970 **(g-i)**, and Cs8g_pb006960 **(j-l)** associated with seedlessness for Satsuma mandarin. The black markers represent seedless varieties, while the red markers represent seeded varieties. The red boxes represent the variations linked with seedlessness. The codes for these varieties correspond to Supplementary Tab. 6.
